# Towards a perfusion system for functional study of membrane proteins with independent control of the electrical and chemical transmembrane potential

**DOI:** 10.1007/s12551-025-01344-4

**Published:** 2025-07-29

**Authors:** Zuzana Coculova, Richard M. Berry

**Affiliations:** 1 University of Oxford, Oxford, UK; 2 UNSW Sydney, Kensington, Australia

**Keywords:** Membrane proteins, Lipid bilayer, Single-molecule functional study

## Abstract

The main motivation of this work was to address the challenge of single-molecule functional study of membrane proteins under stable and independently controlled electrical and chemical membrane potentials. Although transmembrane potential is often essential for the function of membrane proteins, current in vitro systems provide only limited options for studying them under biologically relevant conditions. Our experimental assay is based on the droplet-on-hydrogel bilayer technique (Leptihn et al. Nat Protoc 8:1048–1057, 2013), where a lipid bilayer forms between a sub-millimetre water droplet and a thin hydrogel layer on a glass cover slip, enabling high-resolution microscopy in total internal reflection mode. To extend the application of this assay beyond channels to other membrane proteins, we introduce a custom-built, electronically controlled perfusion system that is designed to directly connect to the droplet above the lipid bilayer. This system can supply a stable voltage to the bilayer and is suitable for delivery of fragile membrane proteins embedded in proteoliposomes via charged fusion (Ishmukhametov et al. Nat Commun 7:13025, 2016), introducing changes of chemical potentials, and timed introduction of labels or substrate into the droplet. This work represents one of the steps towards single-molecule functional study of F_1_F_o_ ATP synthase under variable transmembrane potentials. High-resolution single-molecule observation of its rotation steps on the microsecond timescale could provide valuable insights into the mechanisms of energy transport across the molecule.

**Supplementary Information:**

The online version contains supplementary material available at 10.1007/s12551-025-01344-4.

## Introduction

The function of a protein is determined by its structure. Therefore, the function of proteins incorporated into lipid membranes depends not only on the composition of the solution they are suspended in but also on the lipid environment of the membrane in which they are embedded. A key property of biological membranes is the transmembrane potential. However, many artificial lipid systems used in single-molecule assays do not allow establishment of the transmembrane potential (e.g. nanodiscs, which do not physically separate the solutions on the opposite sides of the membrane) or do not allow for the membrane potential to be stable and easily adjustable (e.g. liposomes, as their inner side is not accessible). Systems that do allow free access to the membrane from both sides traditionally have very limited stability (e.g. suspended membranes).

Our motivation was to develop a platform to allow high-resolution single-molecule observation of functional F_1_F_o_ ATP synthase in a stable energized lipid bilayer. As stated in the review *Rotation and structure of F*_*o*_*F*_*1*_*-ATP synthase* (Okuno, et al. 2011): “Experimental systems that allow stable charging of the membrane potential simultaneously with observation of F_1_ rotation with high spatiotemporal resolution are highly awaited”. Recent developments in this area include arrayed lipid bilayer chambers (ALBiC), a microscale platform developed for quantitative analysis of transporter activity (Watanabe et al. 2014a). ALBiC was used for recording passive proton transport by α-hemolysin and passive proton transport by F_1_F_o_ during ATP hydrolysis. ALBiC can be also used to study membranes with asymmetric lipid composition (Watanabe et al. 2014b) or to create a concentration gradient of target molecules, enabling parallel measurement of multiple bioassays under different conditions (Watanabe et al. 2018). A different approach uses cryogenic electron microscopy to reveal rotational and inhibited substates of F_1_F_o_ with the addition of ADP and ATP (Sobti et al. 2020, 2023). Nonetheless, the question of how energy from proton transport through tenfold symmetric F_o_ is temporarily stored and later used for conformational changes enabling catalysis of ATP in threefold symmetric F_1_ in *E. coli* F_1_F_o_ remains unanswered due to the lack of a suitable experimental method to study this energy conversion in real-time.

We propose that the following series of steps may lead to progress in investigating this question:

1. Designing and building a stable perfusion system with less than 50 nl step size (reported here)
2. Adapting droplet-on-hydrogel bilayer assay for use with perfusion system and show that the droplets are stable in adapted system (reported here)
3. Testing the capability of the perfusion system to not just flow the liquid through the droplet but completely exchange the content of the droplet above the bilayer in order to control chemical and electrochemical potentials
4. Lipid fusion for protein delivery to the bilayer, published (Ishmukhametov et al. 2016), measuring functional activity of F_1_F_o_ in the droplet-on-hydrogel bilayer
5. Stabilizing the F_1_F_o_ stator by attaching it to the hydrogel, published (Ho 2010)
6. Using high-speed polarization microscopy for single-molecule rotation traces of protein motor labelled with gold nanorod, published (Rieu et al. 2025)

### Droplet-on-hydrogel bilayer (DHB)

DHB, as developed by Leptihn et al. (2013), is a planar lipid bilayer supported by a hydrogel film. The support is created by spin-coating a thin layer of hydrogel onto a glass coverslip, which is then covered with an oil-lipid mixture. When a water droplet is introduced into the oil-lipid mixture, it sinks due to its higher density. Lipid monolayers spontaneously form at hydrogel-oil and droplet-oil interfaces. When the droplet contacts the hydrogel, these monolayers fuse to form a lipid bilayer.

DHBs were successfully used to study various ion channels reconstituted into the bilayer from the aqueous phase (Heron et al. 2009; Booth et al. 2017). However, this reconstitution method is not suitable for fragile membrane proteins. We believe that we can overcome this problem by employing a perfusion system to deliver cationic proteoliposomes into the droplet and use charged lipid fusion (Ishmukhametov et al. 2016) to embed membrane proteins into the droplet-on-hydrogel bilayer without significant loss of activity. Other systems allowing buffer exchange on the membrane include the microfluidic platform for forming suspended lipid bilayers published by Zagnoni et al. (2009) and a system with masking apertures used to overcome the problem of rupture of the fragile droplets in the method of Portonovo and Schmidt (2012). Both of these methods, however, only allow the buffer below the bilayer to be exchanged and do not perfuse the droplet. This article describes the steps we take towards this goal.

## Methods

### Preparation of oil-lipid mixture

The glass vials used for preparing the oil-lipid mixture were thoroughly cleaned: each vial was rinsed three times with 2% (v/v) Hellmanex, diluted in ultrapure water (18.2 MΩ·cm, Milli-Q, Sigma-Aldrich), filled with the same solution, and sonicated for 6 min. This procedure was then repeated with ultrapure water, ethanol, and again with ultrapure water. After rinsing, the vials were dried using a nitrogen stream, sealed with lids and parafilm. Cleaned vials could be stored for several months. To prepare the oil-lipid mixture, 1.25 mM of 1-palmitoyl-2-oleoyl-sn-glycero-3-phosphate (POPA^−^, Avanti Research) in chloroform and 3.75 mM of 1,2-diphytanoyl-sn-glycero-3-phosphocholine (DPhPC, Avanti Research) in chloroform were transferred into a cleaned glass vial. The chloroform was evaporated slowly in a fume hood under a gentle nitrogen stream while rotating the vial, leaving a uniform lipid film on the walls. The vial was then placed in a vacuum desiccator for 2 h or overnight to remove residual chloroform. Following this, 0.25 ml of hexadecane and 0.25 ml of silicone oil, both filtered using a 0.22-µm syringe filter, were added, as published in Booth et al. (2016). The mixture was then shaken at 37 °C and 700 rpm for 1.5 h in a shaking incubator.

### Applying agarose layer

We used the protocol for cleaning coverslips published in Veshaguri et al. (2016). 24 × 40 mm #1 coverslips (Menzel-gläser) were sonicated in 2% (v/v) Hellmanex diluted in ultrapure water for 5 min, followed by five rinses with ultrapure water, sonication in ultrapure water for 5 min, five additional rinses with ultrapure water, sonication in ethanol for 5 min, and five more rinses with ultrapure water. The cleaned coverslips were stored in ultrapure water inside sealed containers for several months. Before use, a coverslip was dried using a nitrogen stream. A 1% (w/v) solution of UltraPure low-melting-temperature agarose (Invitrogen) was prepared in ultrapure water and melted at 70 °C and 800 rpm for 15 min in a shaking incubator. The surface of the coverslip was activated via plasma treatment for 7 min at 95 W with a 0.5 bar oxygen stream. Within a few minutes of plasma treatment, the coverslip was placed on a spin coater (Laurell, model no. WS-650MZ-23NPP/LITE). A 140 µl droplet of melted agarose was applied to its centre, and the coverslip was spun at 3000 rpm for 1 min. The spin-coated coverslips were stored in sealed glass containers for several months.

### PMMA microfluidic chip

**Fig. 1:**
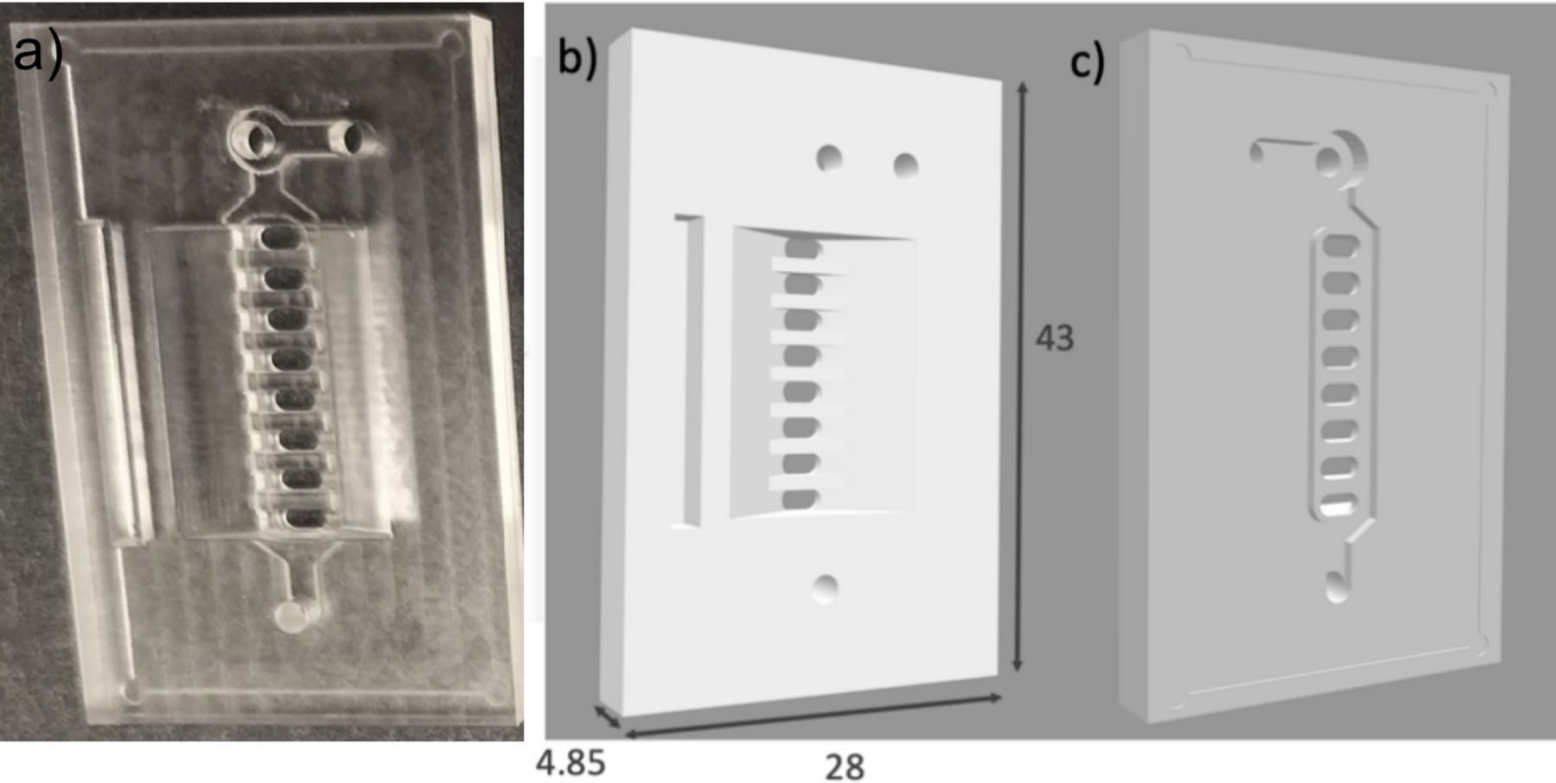
PMMA chip for DHB adapted for use with perfusion system. **a** Photograph of the chip. **b**,** c** 3D model of the chip, top and bottom view, respectively. Dimensions are indicated in millimeters. 3D model is available in Supplementary materials.

We modified the original chip design published by Leptihn et al. (2013) to allow for the insertion of micropipettes into the droplet at a 50° angle relative to the horizontal plane (Fig. 1). The chip was designed using AutoCAD software and fabricated via computer numerical control (CNC) machining from a 43 × 28 × 4.85 mm block of polymethyl methacrylate (PMMA). A 3D model of the chip is available in the Supplementary materials. The chip is compatible with 24 × 40 mm #1 (0.13–0.16 mm thick) coverslip.

### Assembling the chip

This protocol was adapted from Leptihn et al. (2013) with changes in agarose concentration. To assemble the chip, a spin-coated coverslip was placed on the bottom, with the agarose-coated side facing inward. It was secured around its perimeter using a thin strip of tape. A 2% (w/v) solution of UltraPure low-melting-temperature agarose (Invitrogen) was prepared in the same buffer intended for the droplet. The choice of buffer should match the experimental requirements for the protein of interest. The agarose solution was melted at 70 °C and 800 rpm in a shaking incubator for 15 min. With the coverslip facing downward, approximately 100 µl of the melted agarose was slowly injected into the underside cavity of the chip through a single inlet until it began to overflow through the two outlet ports on the opposite side. During this injection, the chip was pressed firmly against the coverslip to ensure even coverage and adhesion. Gelation occurred within about 1 min.

After gelation, the chip was turned over and inspected visually to confirm that the wells were not flooded with agarose—this was verified by checking for reflection from the air/glass interface within a well. The chip was then placed in a high-humidity chamber (an enclosed plastic container with moistened tissue paper) for 15 min to hydrate the agarose layer thoroughly. Finally, the wells were filled with the oil-lipid mixture, allowing monolayer formation at the agarose–oil interface over the next 30 min.

### Preparing the droplets and bilayer formation

This protocol was adapted from Leptihn et al. (2013). At the same time, the wells were filled with the oil-lipid mixture, and an identical solution was added to a separate incubation compartment located on the chip. Using a 2.5-µl syringe, eight 200 nl droplets of buffer were injected into the oil-lipid mixture, ensuring they remained spatially separated to prevent unintended fusion.

**Fig. 2:**
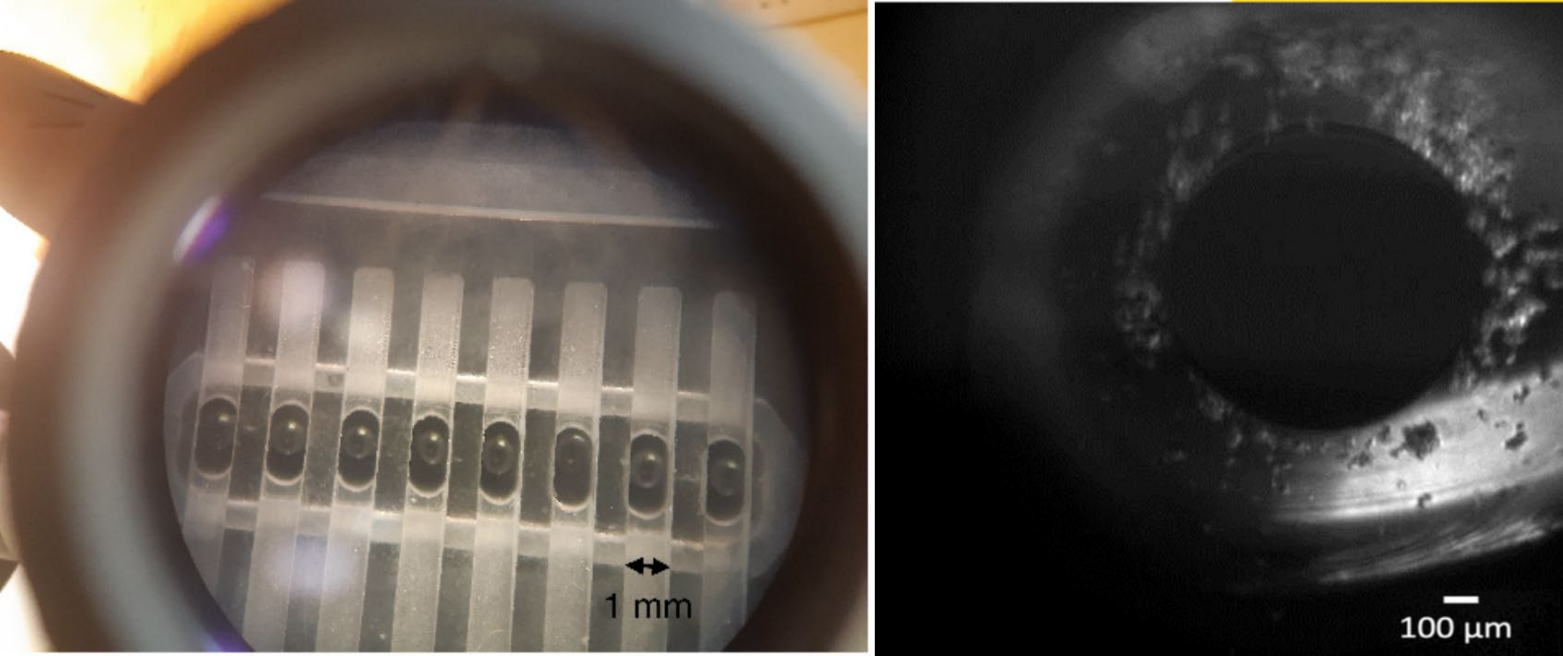
**a** Top view of the assembled chip with a 200 nl droplet (~ 726 μm diameter) in each well, observed under the microscope. **b** Bottom view of a droplet-on-hydrogel bilayer below the droplet under the microscope

After 30 min, lipid monolayers spontaneously formed at the buffer-oil interface of each droplet. The droplets were then carefully transferred into individual wells, along with a small amount of surrounding oil-lipid solution to prevent their exposure to air. Due to their higher density compared to the oil-lipid mixture, the droplets sank to the bottom of the wells. When the monolayer on the droplet surface contacted the monolayer at the hydrogel interface, a planar lipid bilayer was formed between the two (Fig. 2).

### Lipid bilayer composition

Two main criteria guided our choice of lipid bilayer composition: bilayer stability and compatibility with the activity of the target protein. We used a mixture of 75 wt% 1,2-diphytanoyl-sn-glycero-3-phosphocholine (DPhPC)—a neutral lipid with highly branched hydrophobic tails known for forming stable planar membranes—and 25 wt% negatively charged 1-palmitoyl-2-oleoyl-sn-glycero-3-phosphate (POPA^−^). This lipid mixture was dissolved in a 1:1 (v/v) mix of hexadecane and silicone oil. The resulting bilayers were stable for several hours.

### Perfusion system

**Fig. 3:**
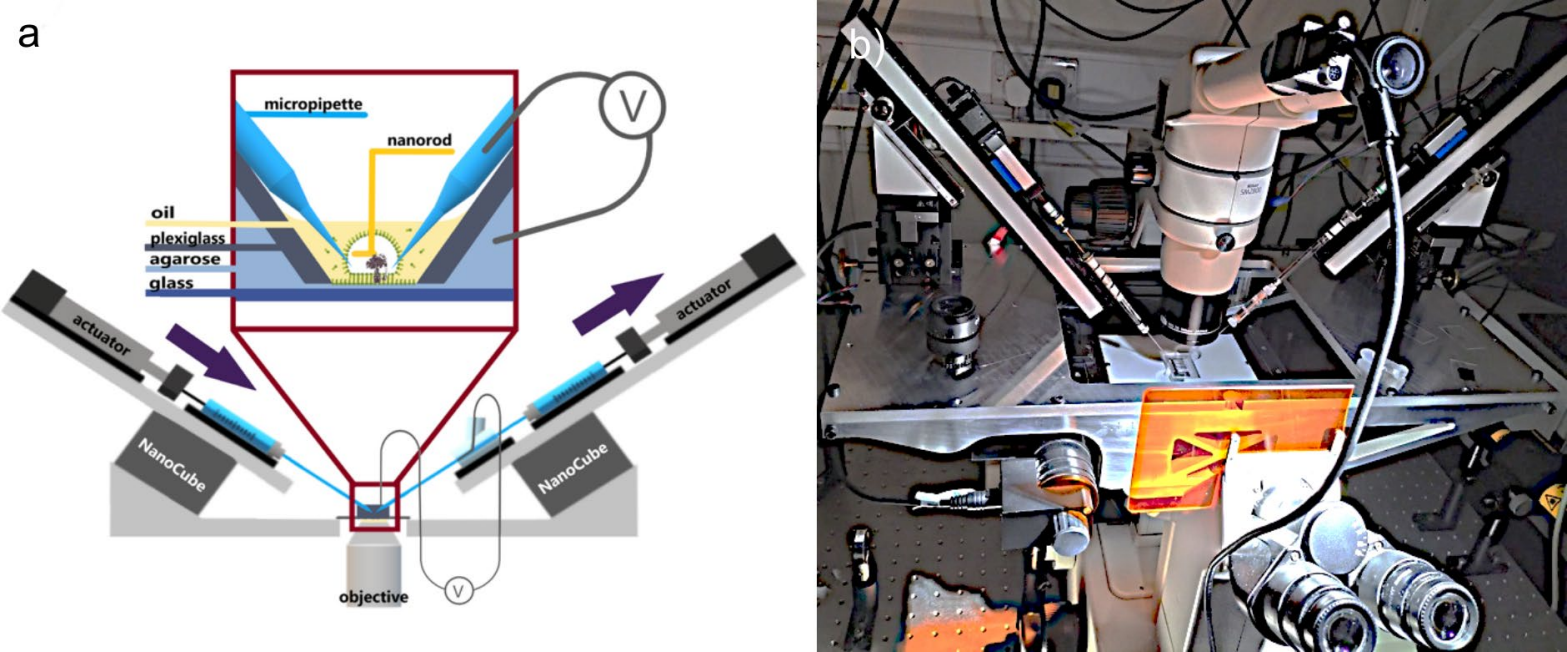
Schematic image of the perfusion system built to perfuse a droplet above droplet-on-hydrogel bilayer (zoomed in and framed in dark red). This system is a step towards the system for high-resolution single-molecule tracking of F_1_F_o_ or other membrane proteins in energized lipid membrane. **b** Photograph of the perfusion system built on a Nikon Eclipse TE200

The primary goal of this work was to extend the applicability of the droplet-on-hydrogel bilayer assay from membrane channels to more fragile membrane proteins. While some ion channels can spontaneously integrate into the bilayer from the aqueous phase and retain their function, transport proteins often require the support of a lipid bilayer to maintain structural integrity. Protein delivery while preserving function can be achieved via charged membrane fusion (Ishmukhametov et al. 2016). In that study, cationic proteoliposomes fused with anionic membranes in giant unilamellar vesicles under low-salt conditions. We propose that the same principle can be applied to planar anionic membranes to facilitate the fusion of proteoliposomes carrying fragile membrane proteins. However, this requires a method for introducing proteoliposomes to a DHB. Injecting material into a 200 nl droplet suspended above a delicate lipid bilayer requires a very stable and precise delivery system. We developed a fully electronically controlled perfusion system that maintains constant droplet volume through synchronized inflow and outflow. Glass micropipettes are connected to Hamilton syringes, whose plungers are driven by stepper-motor actuators. These actuators push and pull in a synchronized manner to prevent changes in droplet volume that might rupture the bilayer (Fig. 3).

Any elasticity or air bubbles in the system could introduce flow delays, volume fluctuations, or droplet rupture. To avoid this, the liquid path was designed to run continuously through rigid glass components—from syringe, through micropipette, into the droplet, and out again via a second micropipette, sturdy electrode holder*,* and syringe—completely sealed from air and elastic materials. This design enabled reliable, precisely synchronized solution exchange inside the droplet. Another possible source of disturbance possibly rupturing the droplet is positioning of the micropipettes. We therefore suspended the entire system on two arms, each of them holding one actuator and one syringe, and each of the arms is placed on a three-axis piezoelectric stage (NanoCube, PI,P-611.3) which is remotely controlled. We mounted a Nikon SMZ800N stereo microscope with a small digital camera (Basler Ace) above the droplet, which was used for navigation of the pipettes’ positioning.

### Electrode holder

**Fig. 4:**
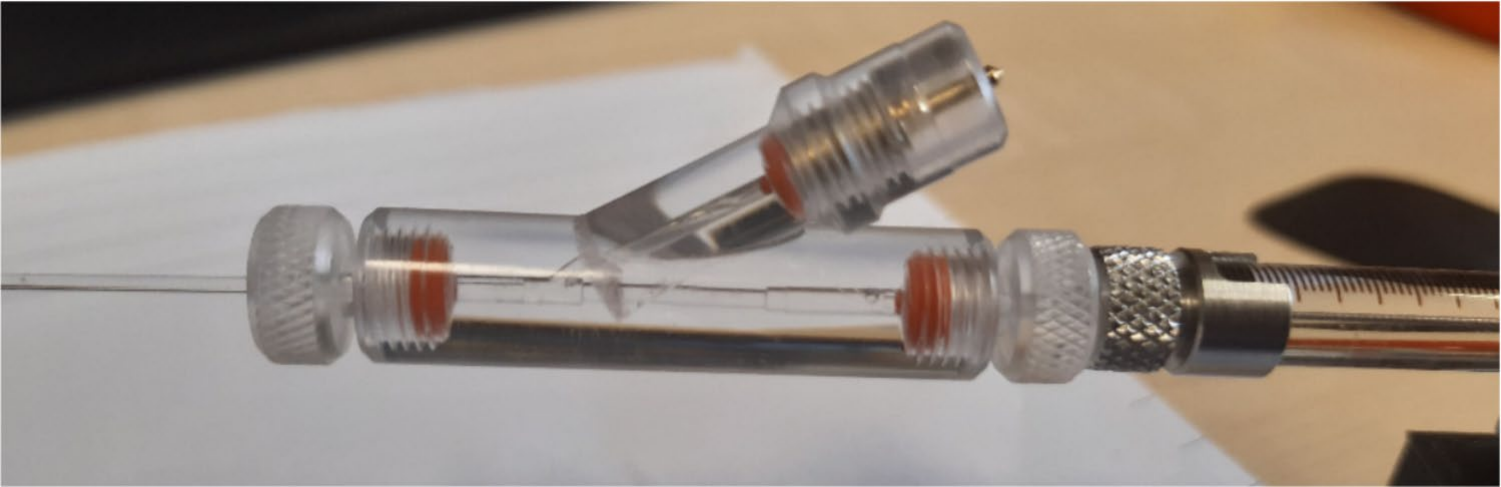
Custom-made polycarbonate electrode holder with Ag/AgCl electrode placed in the arm of the holder. The holder is glued to the outflow syringe

To avoid disturbing the droplet, we placed the electrode not directly into the droplet, but into the liquid connected to it by the perfusion pipettes. The electrode holder was mounted on the outflow side, minimizing the risk of contamination. We used a custom-made polycarbonate holder equipped with a BNC (Bayonet Neill–Concelman) connector and an Ag/AgCl electrode (Fig. 4). Following the protocol published in Leptihn et al. (2013), the silver electrodes were chlorinated by immersion in 20% sodium hypochlorite solution.

### Micropipettes

**Fig. 5:**
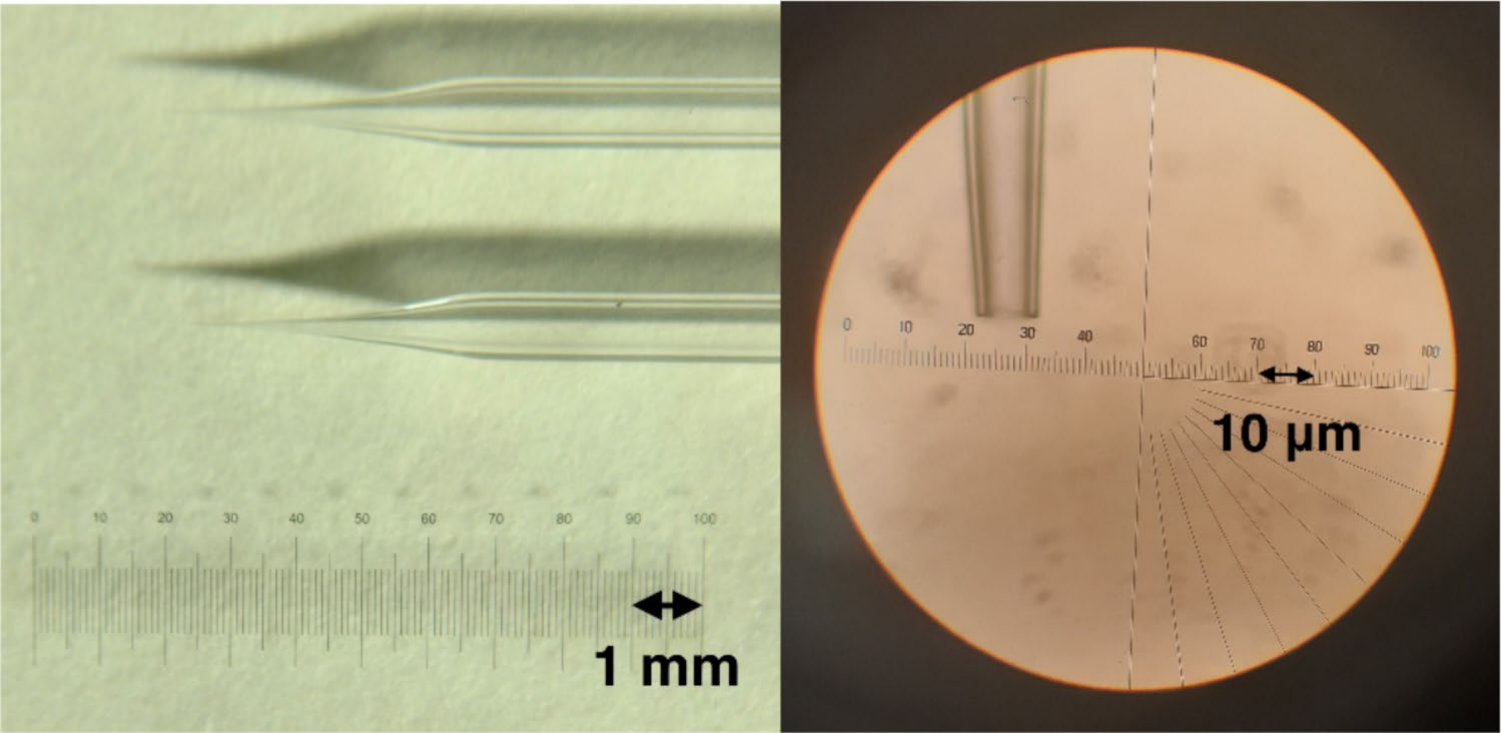
Micropipettes were pulled from borosilicate glass capillaries and hydrophobically coated in Sigmacote. We used approximately 4 mm long and 10 μm thick tips

Micropipettes were fabricated by pulling borosilicate glass capillaries (1B100-3, World Precision Instruments) with an outer diameter of 1 ± 0.1 mm, an inner diameter of 0.58 ± 0.1 mm, and a length of 76 mm using a Sutter Instrument Micropipette Puller. The pipette tips were approximately 4 mm in length and 10 µm in diameter at their ends (Fig. 5). Tips were hydrophobically treated by dipping them in Sigmacote, allowing them to dry completely, and repeating this process twice. The micropipettes were attached to Hamilton syringes using removable needle compression fittings.

### Actuators

In comparing actuators with permanent magnet direct current (PMDC) motors and stepper motors, we found stepper motors more suitable for this application because their movement speed is independent of load. We used Actuonix S20 linear stepper actuators, each equipped with a hybrid bipolar stepper motor NEMA8 (National Electrical Manufactures Association) with a step size of 1.8° and an arm length of 50 mm. The actuators were controlled using custom LabVIEW software, using a National Instruments NI-9263 voltage output module and a current amplifying circuit with quadruple high-current half-H driver L293D for each actuator.

### Platform

The perfusion system was built on a Nikon Eclipse TE200 inverted microscope with removed condenser. The chip was mounted on a motorized microscope stage, while the remaining components of the perfusion system were secured directly to the microscope body, static relative to the microscope objective. Although not strictly necessary, this configuration simplified moving from one droplet to another one. Instead of repositioning the micropipettes for each droplet, we kept them fixed just above the oil surface and moved the microscope stage to target different wells.

### Rod

**Fig. 6:**
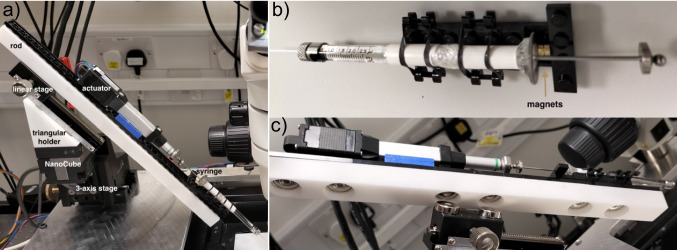
**a** Inflow part of the perfusion system: actuator and syringe are secured on a single rod, which position is controlled by a system of stages. **b** Syringe holder is equipped with neodymium magnets glued to the Lego structure. **c** Rod is outfitted with Lego pieces for stable and versatile mounting and secured to the linear stage by a pair of neodymium magnets

The support platform holds two Thorlabs three-axis manual translation stages, symmetrically positioned on either side of the objective. Each stage supported a three-axis piezoelectric stage (NanoCube, PI, P-611.3) for fine positioning. A 3D-printed triangular adapter connected each NanoCube to a manual dovetail rack-and-pinion linear stage, with the translation axis orientated along the micropipettes. We used neodymium magnets to attach a 3D-printed rod to the linear stage. The top of the rod was outfitted with LEGO pieces, providing a stable and modular mounting platform for the actuator and syringe. Special attention was paid to firmly anchoring both the syringe and the actuator arm. In early versions of the setup, even slight movements of the syringe resulted in flow inconsistencies. The use of magnets ensured a secure yet easily removable connection, reducing the risk of misalignment or droplet rupture (Fig. 6).

## Results

**Fig. 7:**
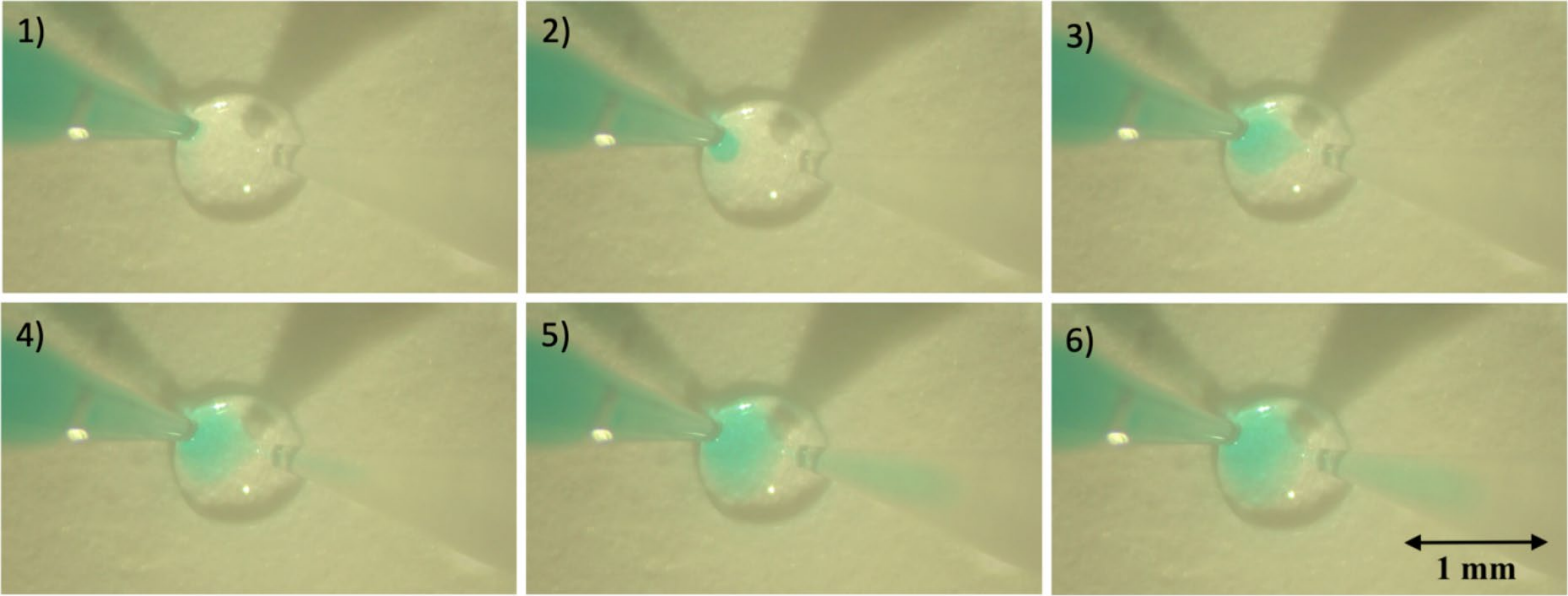
Visualisation of the perfusion of a 200 nl water droplet on a glass surface with coloured water delivered in 27 nl steps. The diameter of the droplet remained constant

We first demonstrated the functionality of our perfusion system by injecting water coloured with food dye into a 200 nl water droplet placed on a glass surface. The inlet pipette on the left delivered the coloured water into the droplet, while the outlet pipette simultaneously withdrew liquid in a synchronized manner. The diameter of the droplet remained constant during the perfusion (Fig. 7). The step size of each injection was 27 nl.

**Fig. 8:**
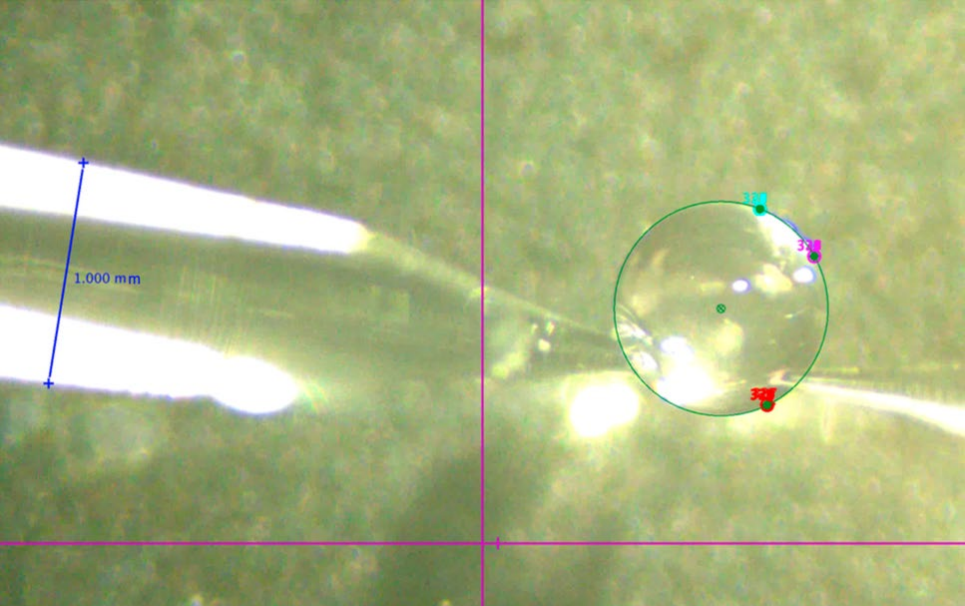
Perfusion of a 200 nl droplet in the air. The volume of the droplet over time was calculated using Tracker. At least three points on the surface of the droplet in every frame were fitted by a circle (green). The blue line defines the scale

To quantitatively evaluate the system’s performance, we conducted experiments using a water droplet suspended in air between an inflow and an outflow pipette. To analyse volume changes during perfusion, we tracked the droplet surface using the Tracker software by D. Brown, W. Christian, and R. M. Hanson. For each droplet, we selected at least three distinct surface points and recorded their positions throughout the experiment (Fig. 8). A circle was then fitted to these points, and the radius was used to calculate droplet volume. The pipette diameter served as the reference for scale.

**Fig. 9:**
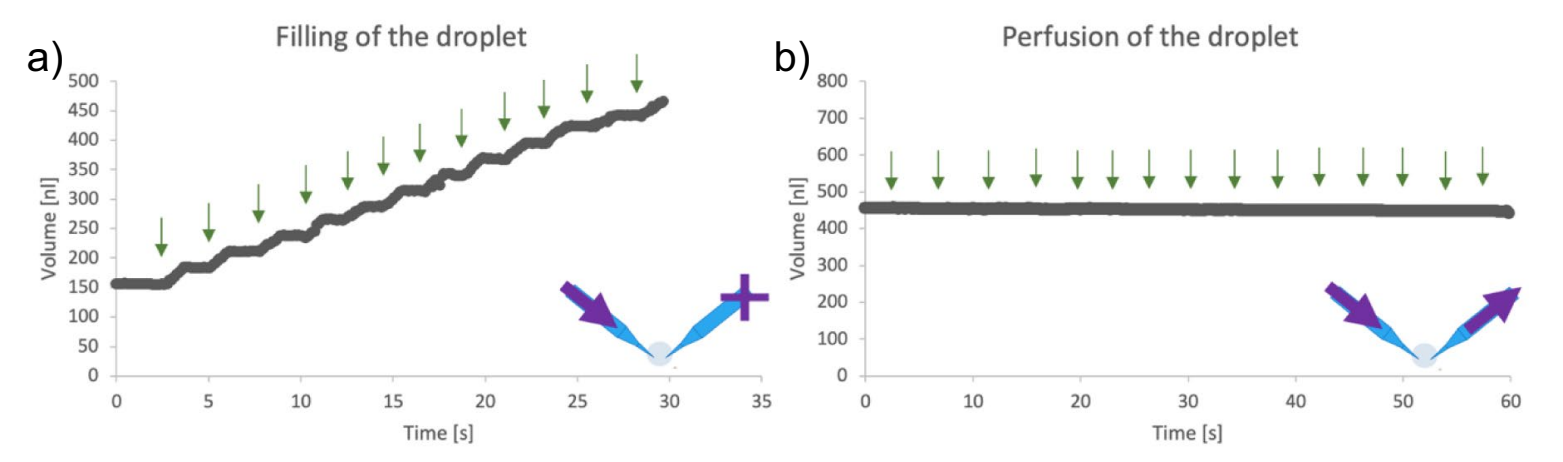
Graph of the volume of the droplet **a** filled through the inflow pipette stepwise at the times indicated by green arrows—average step size was 26.68 ± 2.5 nl, and **b** perfused through inflow and outflow pipettes stepwise at the times indicated by green arrows. The volume was fitted by a line, and the standard deviation of the volume was 0.70 nl

Figure 9a shows the droplet volume increasing over time when only the inflow pipette was engaged. Water was injected in discrete steps, indicated by green arrows on the graph. The outflow pipette was attached but not activated. The average step size was 26.68 nl with a standard deviation of 2.5 nl. Figure 9b shows an example of an experiment in which both pipettes were engaged, resulting in synchronized inflow and outflow. The timing of each perfusion step is marked with green arrows. It shows a stable, consistent perfusion without significant disturbance to the droplet. We fitted the volume trace with a linear trend line and the standard deviation of the droplet volume was 0.70 nl.

If the droplet is perfused with a solution of different ionic concentration than that of the hydrogel beneath it, passive diffusion through the membrane may cause a dissipation of the chemical potential. The hydrogel volume is approximately 500 times greater than that of the droplet, which should, together with the ion mobility in the hydrogel, be taken into account when estimating how long the assumption of a constant ionic concentration on the hydrogel side remains valid.

## Conclusion

We have developed a custom-built perfusion system that has the potential to extend the capabilities of the droplet-on-hydrogel bilayer assay. Traditionally used for studying highly robust ion channels, this assay can be adapted for the functional study of fragile membrane proteins—such as F_1_F_o_ ATP synthase—under precisely controlled electrical and chemical potentials, with direct access to the droplet for the delivery of ligands, labels, or substrates.

Quantitative analysis using Tracker software demonstrated that the perfusion system reliably perfuses droplets as small as 726 µm in diameter. The minimal variations observed in droplet volume were likely due to a combination of image processing limitations, minor mechanical disturbances, and evaporation. However, these fluctuations were so small that they would not have the potential to rupture the droplet. The experimentally determined step size during injection when the outlet pipette was not engaged was 26.68 ± 2.5 nl.

We believe that this system is one of the steps towards recording high-resolution rotation traces of F_1_F_o_ during ATP synthesis. Such data could provide critical insight into the long-standing question of how energy from proton transport is temporarily stored and subsequently used to drive conformational changes during ATP catalysis.

Beyond F_1_F_o_, such a system could be used for the study of other ion-driven molecular motors—including bacterial 5:2 rotary motors, whose rotation has never been directly observed (Rieu et al. 2022)—and open new experimental possibilities for single-molecule studies in controlled membrane environments.

## Supplementary Information

Below is the link to the electronic supplementary material.

ESM 1 (STL 97.2 KB)

## Data Availability

No datasets were generated or analysed during the current study.
